# Metabolism disorder promotes isoproterenol-induced myocardial injury in mice with high temperature and high humidity and high-fat diet

**DOI:** 10.1186/s12872-022-02583-z

**Published:** 2022-03-30

**Authors:** Taohua Lan, Qiaohuang Zeng, Wei Jiang, Tong Liu, Wenjing Xu, Ping Yao, Weihui Lu

**Affiliations:** 1 State Key Laboratory of Dampness Syndrome of Chinese Medicine, The Second Affiliated Hospital of Guangzhou University of Chinese Medicine, Guangzhou, 510020 People’s Republic of China; 2 Guangdong Provincial Key Laboratory of Chinese Medicine for Prevention and Treatment of Refractory Chronic Diseases, Guangzhou, 510020 People’s Republic of China; 3 Guangdong Provincial Hospital of Chinese Medicine, Guangzhou, 510020 People’s Republic of China; 4 Guangdong Provincial Academy of Chinese Medical Sciences, Guangzhou, 510020 People’s Republic of China; 5 Department of Cardiology, Guangdong Provincial Hospital of Chinese Medicine, No. 111, Dade Road, Yuexiu District, Guangzhou, 510020 People’s Republic of China

**Keywords:** Isoproterenol, Myocardial injury, Phlegm-damp syndrome, Metabolism disorder, High temperature, High Humidity, High-fat diet

## Abstract

**Background:**

Isoproterenol (ISO), a synthetic on selective β-adrenergic agonist, provides a simple and non-invasive method for inducing myocardial injury with lower mortality and higher reproducibility. Phlegm-damp syndrome, as known as “Tanshi” in Chinese, is one of Traditional Chinese Medicine (TCM) syndrome differentiation, which plays an important role in the development of cardiovascular diseases. However, the underlying mechanism remains unknown.

**Methods:**

In our present study, a myocardial injury mouse model was introduced by ISO administration combined with high temperature and high humidity and high-fat diet to simulate phlegm-damp syndrome. Nontargeted metabolomics with LC–MS/MS was adopted to reveal serum metabolism profile for elucidating the possible molecular mechanism.

**Results:**

The results of our study showed that phlegm-damp syndrome promoted ISO-induced myocardial injury by aggravating left ventricular hypertrophy and fibrosis, and increasing cardiac index. Our study also confirmed the presence of specific metabolites and disturbed metabolic pathways by comparing ISO mice and Tanshi mice, mainly including glycerophospholipid metabolism, arginine–proline metabolism, and sphingolipid signaling pathway. The lysoPCs, PCs, SMs, Sphingosine, and L-Arginine were the main metabolites that showed a difference between ISO and Tanshi mice, which might be the result of the underlying mechanism in the promotion of ISO-induced myocardial injury in mice with high temperature and high humidity and high-fat diet.

**Conclusion:**

Our current study provides new insights into contribution of metabolism disorder in promotion of ISO-induced myocardial injury in mice with high temperature and high humidity and high-fat diet, and new targets for clinical diagnosis and pharmacologic treatment of cardiovascular disease with phlegm-damp syndrome.

**Supplementary Information:**

The online version contains supplementary material available at 10.1186/s12872-022-02583-z.

## Background

Cardiovascular diseases (CVDs), the leading cause of death in humans, have emerged as a high socio-economic burden around the world with rising incidence. It is predicted that CVDs may increase by approximately 21.3 million events and 7.7 million deaths over 2010–2030 in China. Isoproterenol (ISO), a synthetic on selective β-adrenergic agonist, is widely used for inducing experimental CVDs such as myocardial ischemia, hypertrophy and infarction, cardiac fibrosis, and heart failure. Stimulation with ISO leads to the development of oxidative stress, calcium overload, myocardial inflammation and renin–angiotensin release, which ultimately cause CVDs [1]. ISO-induced myocardial injury animal models are reported to be used for the evaluation of cardioprotective agents due to their advantages in lower mortality and higher reproducibility compared with other animal models [2].

Environmental risk factors such as climatic change, are becoming a major public health concern, with increasing studies shown that extreme temperatures and humidity are associated with higher risks of mortality throughout the world [3–5]. Hyperlipidemia, characterized by high serum lipids, is one of the well established risk factors of cardiovascular diseases. Phlegm-damp syndrome, as known as “Tanshi” in Chinese, is one of Traditional Chinese Medicine (TCM) syndrome differentiation, which has the highest incidence in Southern China. Phlegm-damp syndrome is characterized by hyperlipidemia combined with high temperature and high humidity in TCM. Previous researches have revealed the important role of phlegm-damp syndrome in the development of CVDs. However, the underlying mechanism remains unknown.

Metabolomics is a powerful approach for identification and quantification of small molecule metabolites that reflect molecular processes more proximal to disease states. Nontargeted metabolomics is commonly employed to capture the complexity of metabolic networks and reveal novel molecular alterations for its comprehensive analysis of the metabolome. Metabolomics received increasing attention in CVDs research for its help to better explain the biological mechanisms and identify novel biomarkers of CVDs [6]. In present study, nontargeted metabolomics with LC–MS/MS was adopted to reveal serum metabolism profile in ISO-induced myocardial ischemia mice with high temperature high humidity and high-fat diet.

## Methods

### Animal model and grouping

**Fig. 1:**
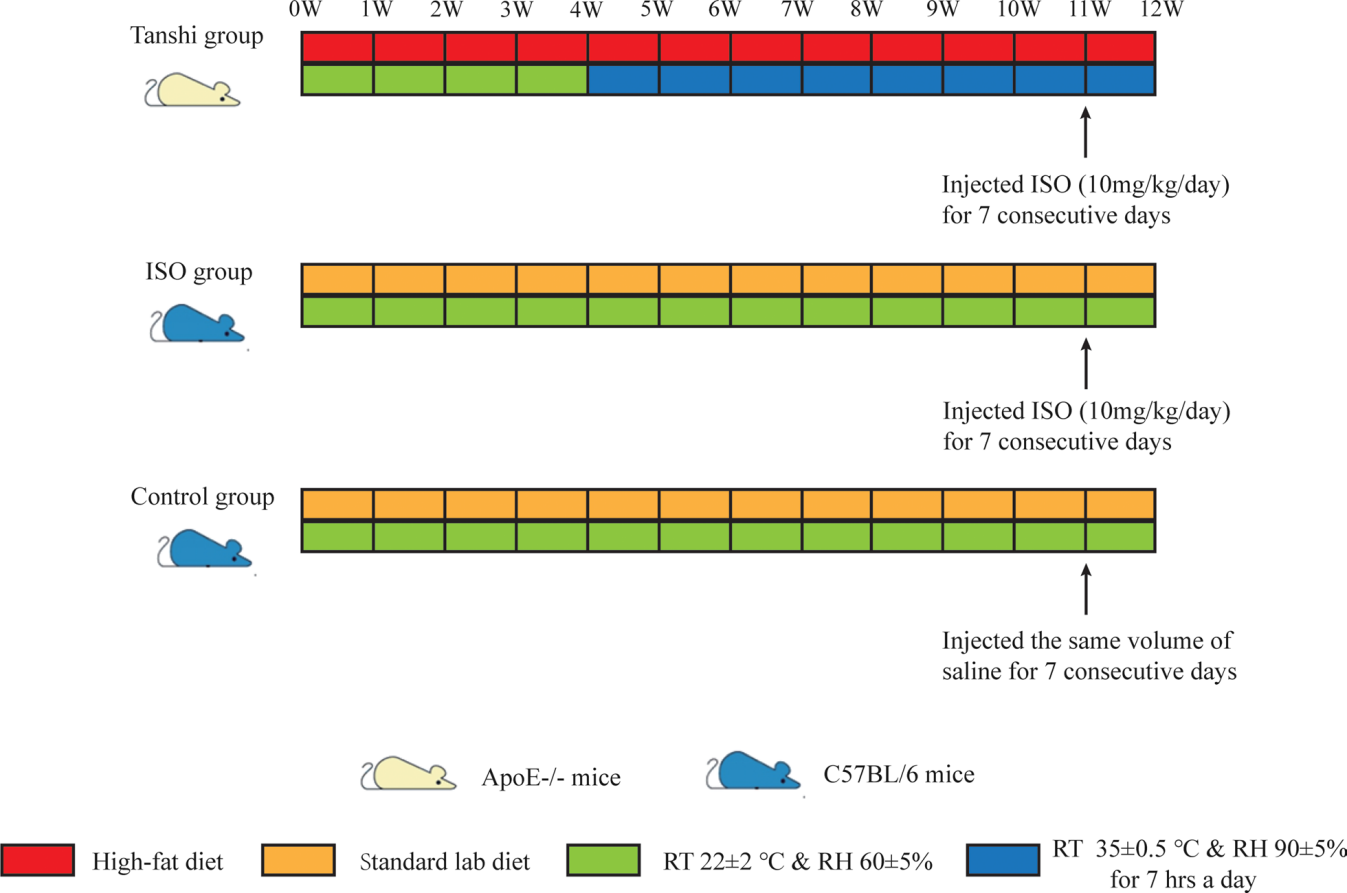
Workflow of grouping and interventions

Male eight-week-old C57BL/6 mice and ApoE−/− mice were obtained from the Beijing HFK Bioscience CO., LTD. The mice were housed under standard conditions with 12 h light/dark cycles and constant room temperature of 22 ± 2 °C and relative humidity of 60 ± 5%, and were fed with standard lab diet (18% protein, 58% carbohydrate, 4.5% fat) for one week prior to further experiments. The animal experiment was approved by the Animal Care and Use Committee of Guangdong Provincial Hospital of Chinese medicine. ApoE−/− mice were fed with high-fat diet that mimics western diet (17.5% protein, 48.5% carbohydrate, 21% fat, 1.5% cholesterol) for 12 weeks as Tanshi group. The standard lab diet and high-fat diet were manufactured by Guangdong Medical Laboratory Animal Center (Guangdong, China). After 4 weeks of high-fat diet administration, ApoE−/− mice were kept in room temperature of 35 ± 0.5 °C and relative humidity of 90 ± 5% for 7 h a day (the rest of day in room temperature of 22 ± 2 °C and relative humidity of 60 ± 5%) and fed with high-fat diet for another 8 weeks. C57BL/6 mice were randomized into control group and ISO group. All C57BL/6 mice were housed under standard conditions with room temperature of 22 ± 2 °C and relative humidity of 60 ± 5% and fed with standard lab diet for 12 weeks. ApoE−/− mice and C57BL/6 mice in ISO group were subcutaneously injected with Isoproterenol (ISO, 10 mg/kg/day) (Sigma, Saint Louis, USA) for seven days to induce experimental myocardial ischemia before sacrifice, while the control mice were given the same volume of saline. A workflow of grouping and interventions was showed in Fig. 1.

### Echocardiography

The echocardiography was performed 2 h after the last ISO injection by using Visual Sonics Vevo 2100 system (Visualsonics Inc., Toronto, Canada) equipped with a 21-MHz linear array transducer. Left ventricular posterior wall thickness (LVPW) and inter ventricular septal thickness (IVS) were measured in Parasternal short-axis view at the level of the papillary muscle by M-mode tracing method.

### Measurement of TC, TG, HDL-C and LDL-C levels in serum

Bloods were collected after echocardiography and centrifuged at 1000 × g for 15 min at 4 °C after 1 h at room temperature. Serum was separated and stored at − 80 °C for assay. The levels of TC, TG, HDL-C and LDL-C in serum were detected using TC Assay Kit (Cat. A111-1-1), TG Assay Kit (Cat. A110-1-1), HDL-C Assay Kit (Cat. A112-1-1), LDL-C Assay Kit (Cat. A113-1-1) manufactured by Nanjing Jiancheng Bioengineering Institute (Jiangsu, China).

### Histomorphology

Mouse hearts were isolated, and perfused with cold saline until without blood in lavage fluid. Cross section of hearts at the level of the papillary muscle were stored in 4% paraformaldehyde for more than 24 h and then embedded in paraffin wax. Tissue blocks were sectioned to 3.5 µm in thickness and stained with hematoxylin and eosin (H&E) or Masson’s trichrome staining to assess left ventricular fibrosis and infarct size according to standard protocols.

### Nontargeted LC–MS-based metabolic profiling

Bloods were collected in 1.5 EP tube and centrifuged at 1000 × g for 15 min at 4 °C after 1 h at room temperature. Serum was separated and stored in − 80 °C for assay. Metabolites were detected in the serum samples from the three groups (n = 6) by LC/MS, as previously described [7]. Serum samples were prepared by thawing at room temperature and 50 µL serum was added to 200 µL methanol and vortexed rigorously before centrifuged at 500 × g for 10 min at 4 °C. Then, the extract was lyophilized and stored at − 80 °C. The dry pellets were reconstituted with sample solvent (water: methanol, 80:20, v/v) and further analysed by liquid chromatography–mass spectrometry (LC–MS) system. QC samples were prepared by mixing aliquots of all samples to be a pooled sample. A Dionex Ultimate 3000 UHPLC (Thermo Fisher Scientific, Waltham, MA, USA) was coupled to Q Exactive plus mass spectrometer (Thermo Fisher Scientific, Waltham, MA, USA) was used to analyze the metabolic profiling. An ACQUITY UPLCHSS T3 column (100 mm × 2.1 mm, 1.8 um; Waters, Milford, MA, USA) was used for metabolite separation. Water and Acetonitrile/Methanol 2/3(v/v), both containing 0.1% formic acid were used as mobile phases A and B, respectively. Linear gradient: 0.0–1.0 min at 5% B; 1–12 min from 5% B to 100% B and keep for 4 min; 16–16.1 min back to 5% B and 16.1–18 min at 5% B. The flow rate was 0.35 mL/min and column temperature was 50 °C. All the samples were kept at 4 °C during the analysis. The injection volume was 2 μL. Data acquisition was performed in full scan mode ranging from 100 to 1000 (m/z) with a resolution of 70,000 for MS1 and resolution17,500 for MS2 was applied. Spray voltages (V) were set at 3800 for positive ionization mode and 3500 for negative ionization mode. Sheath gas and auxiliary gas flow rates were set at 35 and 35 arbitrary, respectively. Capillary and auxiliary gas heater temperatures were set at 320 °C and 320 °C.

### Statistical analysis

For metabolomics analyses [8, 9], principle component analysis (PCA) and partial least-squares-discriminant analysis (PLS-DA) were carried out to visualize the metabolic alterations among experimental groups (ropls package, R sofeware, ver3.6.2). A heatmap was showed with red and blue indicating high and low concentrations, respectively (pheatmap package, R sofeware, ver3.6.2). Metabolite-associated pathways were analyzed using KEGG Database (https://www.kegg.jp/). The differential metabolites were selected on the basis of the combination of a statistically significant threshold of variable influence on projection (VIP) values obtained from the OPLS-DA model and p values from a two-tailed Student’s t test on the normalized peak areas, where metabolites with VIP > 1.0 and *P* < 0.05 were considered as differential metabolites.

For in vivo data, results were expressed as the mean ± S.D. Statistical comparison among multiple groups was performed by one-way ANOVA followed by LSD test using the SPSS 19.0 software. A value of *P *< 0.05 was considered statistically significant.

## Results

### High-fat diet with high temperature and high humidity promotes myocardial injury in ISO-induced myocardial injury mice

**Fig. 2:**
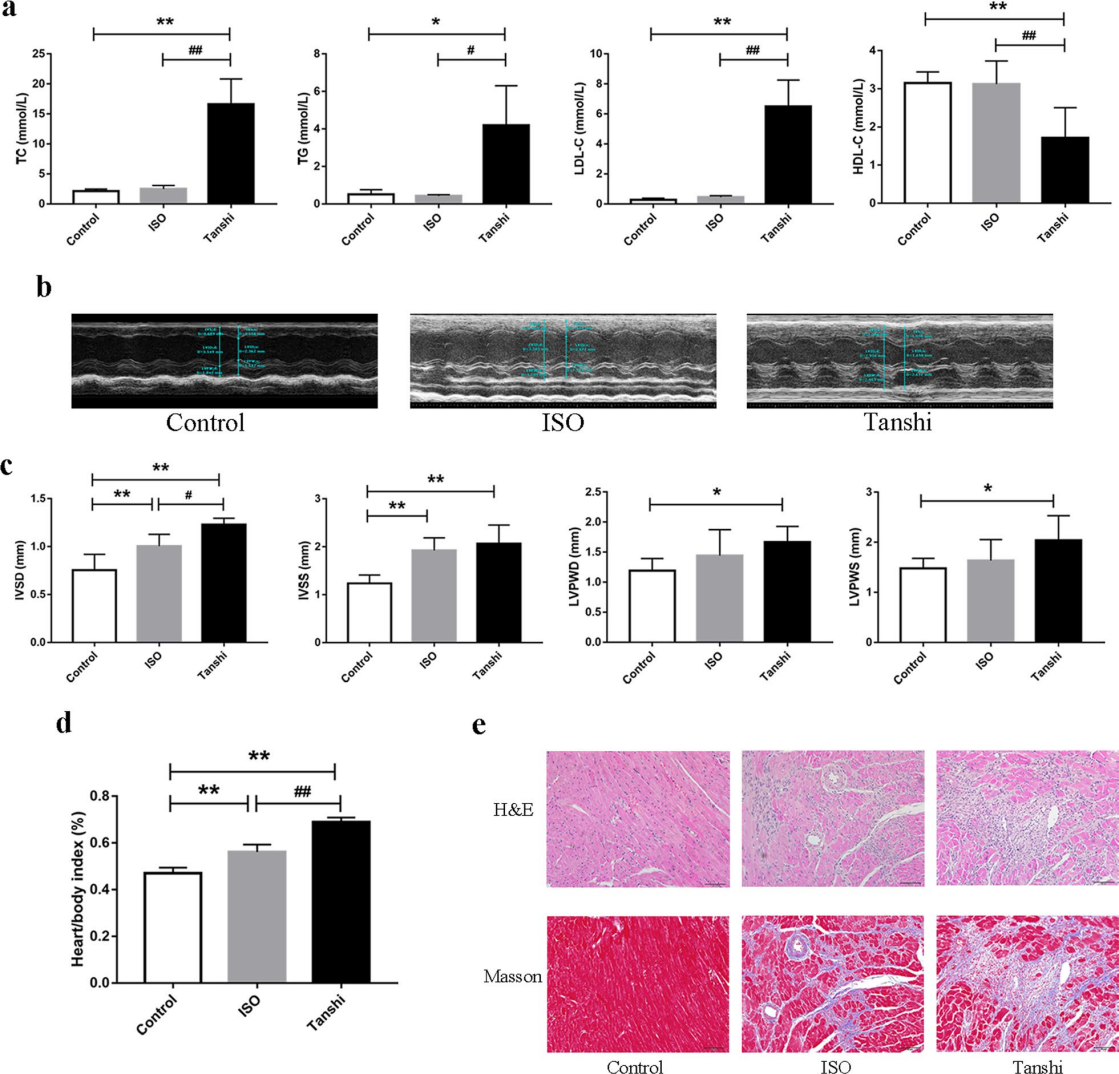
Phlegm-damp syndrome promotes myocardial injury in ISO-induced myocardial injury mice. **a** Serums were collected to evaluate the level of TC, TG, HDL-C and LDL-C. Data are shown as mean ± S.D. (n = 5–6). **b** Left ventricular posterior wall thickness (LVPW) and inter ventricular septal thickness (IVS) was measured by echocardiography and shown as mean ± S.D (n = 5) in (**c**). **d** Cardiac index for each mouse was calculated using the following formula: heart weight/bodyweight × 100%. Data are shown as mean ± S.D (n = 6). **e** The pathological changes and fibrosis of myocardium were assessed by H&E and Masson staining (scale bar = 50 µm). *P < 0.05 versus control group, **P < 0.01 versus control group, ##*P* < 0.01 versus ISO group

As showed in Fig. 2a, the levels of TC, TG and LDL-C in serum of Tanshi mice were significantly higher while HDL-C levels were significantly lower than that of control mice and ISO mice. The results suggested that high-fat diet induced hyperlipemia in Tanshi mice. As showed in Fig. 2b and c, IVSS and IVSD significantly increased in mice of ISO group and Tanshi group compared with control mice (*P* < 0.01). Mice in Tanshi group also showed a significant elevation in LVPWS and LVPWD value compared with the control group (*P* < 0.05). All these values were worse in Tanshi mice compared with the ISO mice. The results suggested that high-fat diet with high temperature and high humidity promotes left ventricular hypertrophy in ISO-induced myocardial injury mice.

After echocardiography measurement and blood collection, the hearts were removed quickly, and cardiac index for each mouse was calculated using the following formula: heart weight /bodyweight × 100%. As showed in Fig. 2d, mice in ISO group developed a significantly increase in cardiac index compared with control mice (*P* < 0.01), and further increase was found in Tanshi mice (*P* < 0.01). Additionally, there was no significant difference in body weight among three groups (as shown in Additional file 1: Fig. S1). These results suggested that high-fat diet with high temperature and high humidity increases cardiac index in ISO-induced myocardial injury mice.

Hematoxylin & eosin (H&E) and Masson’s trichrome staining were used to assess pathological changes and fibrosis of myocardium. As shown in Fig. 2e, myocardial cells in control group had normal cellular morphology and structure without inflammatory cell infiltration. The structure of myocardial cells in model group was disorganized with obvious focal necrosis, and myocardial fibers extensive swelled and ruptured. The pathological changes of myocardium were even worse in Tanshi mice. In addition, a significantly more server myocardial fibrosis was found in ISO mice compared with control mice, which was even worse in Tanshi mice. The results suggested that high-fat diet with high temperature and high humidity aggravates pathological changes and fibrosis of myocardium in ISO-induced myocardial injury mice.

### Serum metabolism profile alters in ISO-induced myocardial injury mice with high temperature and high humidity and high-fat diet

**Fig. 3:**
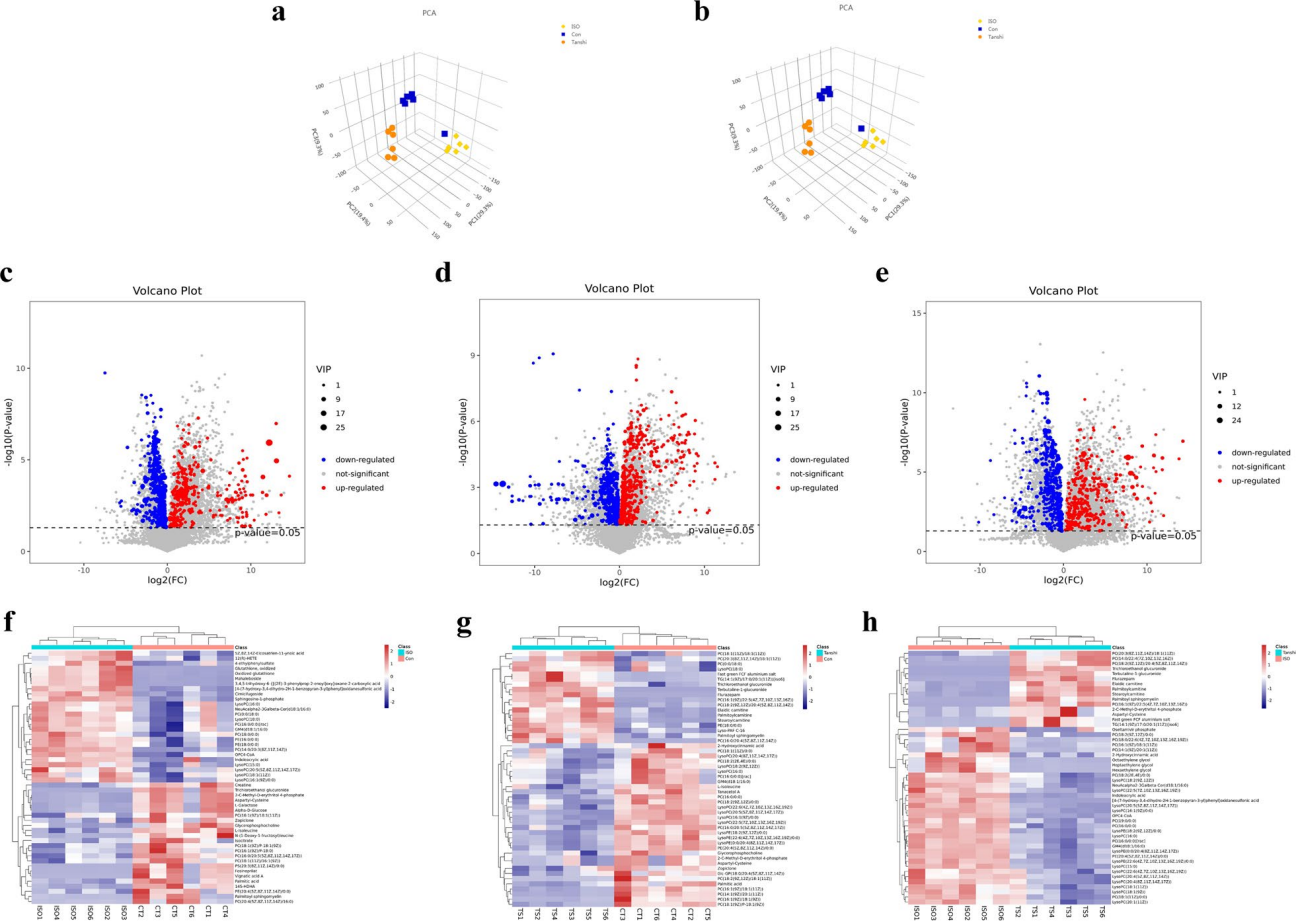
Serum metabolism profile alters in ISO-induced myocardial injury mice with high temperature and high humidity and high-fat diet. **a** Score plots of principle component analysis (PCA). **b** Score plots of partial least squares discriminant analysis (PLS-DA). **c** Volcano plot of differential metabolites in Tanshi group compare with Con group. **d** Volcano plot of differential metabolites in ISO group compare with Con group. **e** Volcano plot of differential metabolites in Tanshi group compare with ISO group. **f–h** Heatmap of differential metabolites in ISO versus Con group, Tanshi versus Con group and Tanshi versus ISO group

PCA and PLS-DA score plots were generated, and the permutation test showed that the models were reliable without overfitting (R2 = (0.0, 0.672), Q2 = (0.0, − 0.973)) in the discovery set. As shown in Fig. 3a and b, the 3-dimensionalscore plot of PCA and PLS-DA proved the apparent separations among control, ISO and Tanshi group, implying obvious changes of the serum metabolism profile in ISO and Tanshi mice.

The Volcano Plots showed 267 and 205 metabolites respectively from Tanshi group (Fig. 3c) and ISO group (Fig. 3d) were significantly up-regulated, while 228 and 215 metabolites were significantly down-regulated compare with control group (p < 0.05). Additionally, 200 metabolites were significantly up-regulated, while 211 metabolites were significantly down-regulated in Tanshi group compare with ISO group (*p* < 0.05) (Fig. 3e). The relative concentrations of the top 50 differential metabolites in all three comparison groups were showed in the heatmaps (Fig. 3f–h), all of which have clear clustering and separation. The top 20 up-regulated and top 20 down-regulated differential metabolites between Tanshi group and ISO group based on P value were shown in Additional file 2: Table S1 and Additional file 3: Table S2, the super class of which was mainly lipids and lipid-like molecules.

**Fig. 4:**
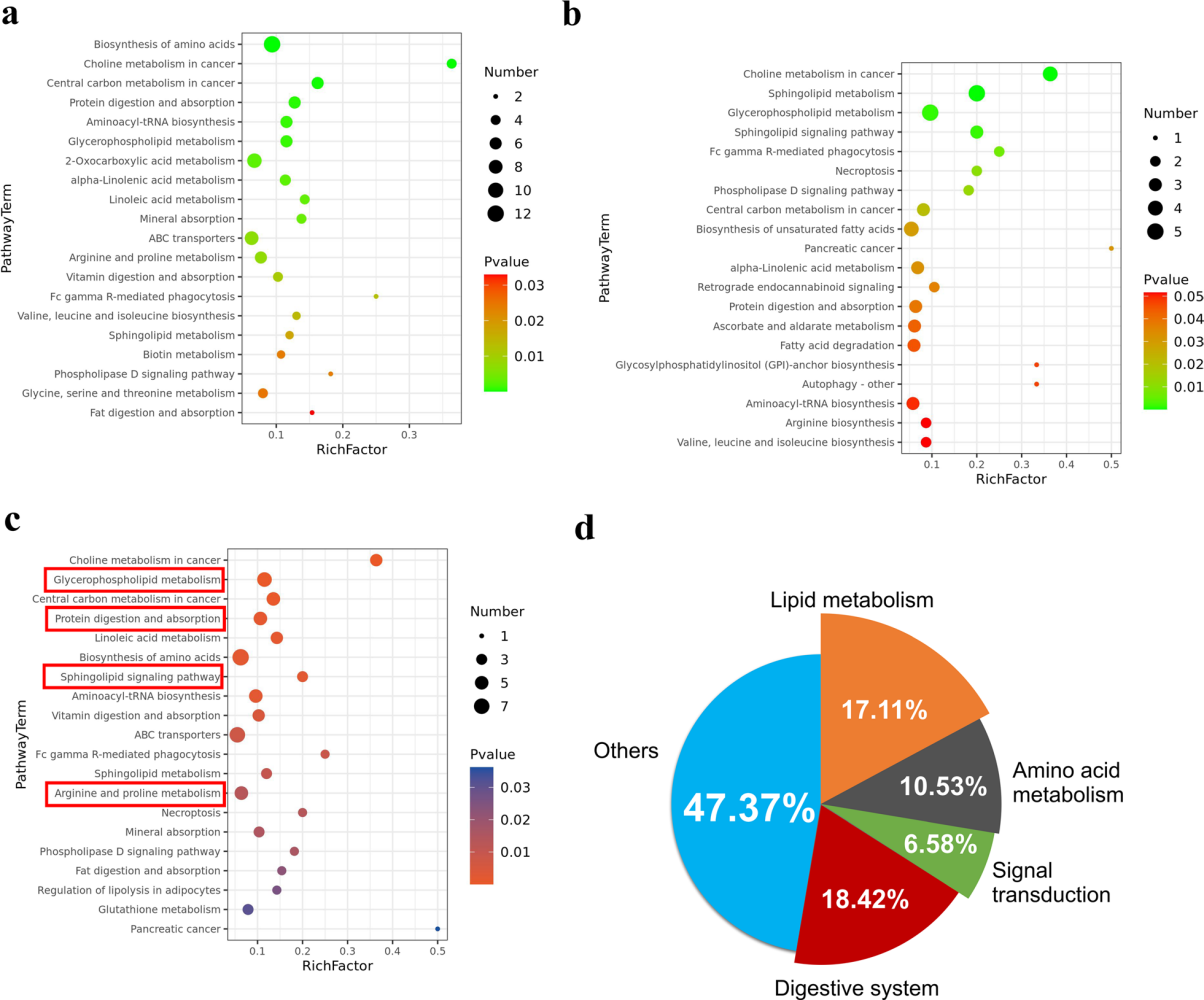
Metabolite-associated pathways. **a** The bubble chart of top 20 KEGG pathways in ISO versus Con group. **b** The bubble chart of top 20 KEGG pathways in Tanshi versus Con group. **d** KEGG subclass of differential metabolites in Tanshi versus ISO group. **c** The bubble chart of top 20 KEGG pathways in Tanshi versus ISO group

To systematically evaluate the perturbed metabolism in all three comparison groups we performed metabolite-associated pathway analyses by using KEGG Database. The top 20 KEGG pathways in ISO versus Con group and Tanshi versus Con group were shown in Fig. 4a and b, indicated that compared with that in control mice, ISO-induced and Tanshi-induced metabolic disturbances shared some of the same pathways while each had own unique pathways. The top 20 KEGG pathways and KEGG subclass of differential metabolites in Tanshi versus ISO group were shown in Fig. 4c and d, implying that compared with that in ISO mice, Tanshi-induced metabolic disturbances were mainly associated with Digestive system (18.42%, e.g., Protein digestion and absorption, Vitamin digestion and absorption, Mineral absorption, Fat digestion and absorption), Lipid metabolism (17.11%, e.g., Glycerophospholipid metabolism, Linoleic acid metabolism, Sphingolipid metabolism), Amino acid metabolism (10.53%, e.g., arginine–proline metabolism), and Signal transduction (6.58%, e.g., Sphingolipid signaling pathway, Phospholipase D signaling pathway).

**Fig. 5:**
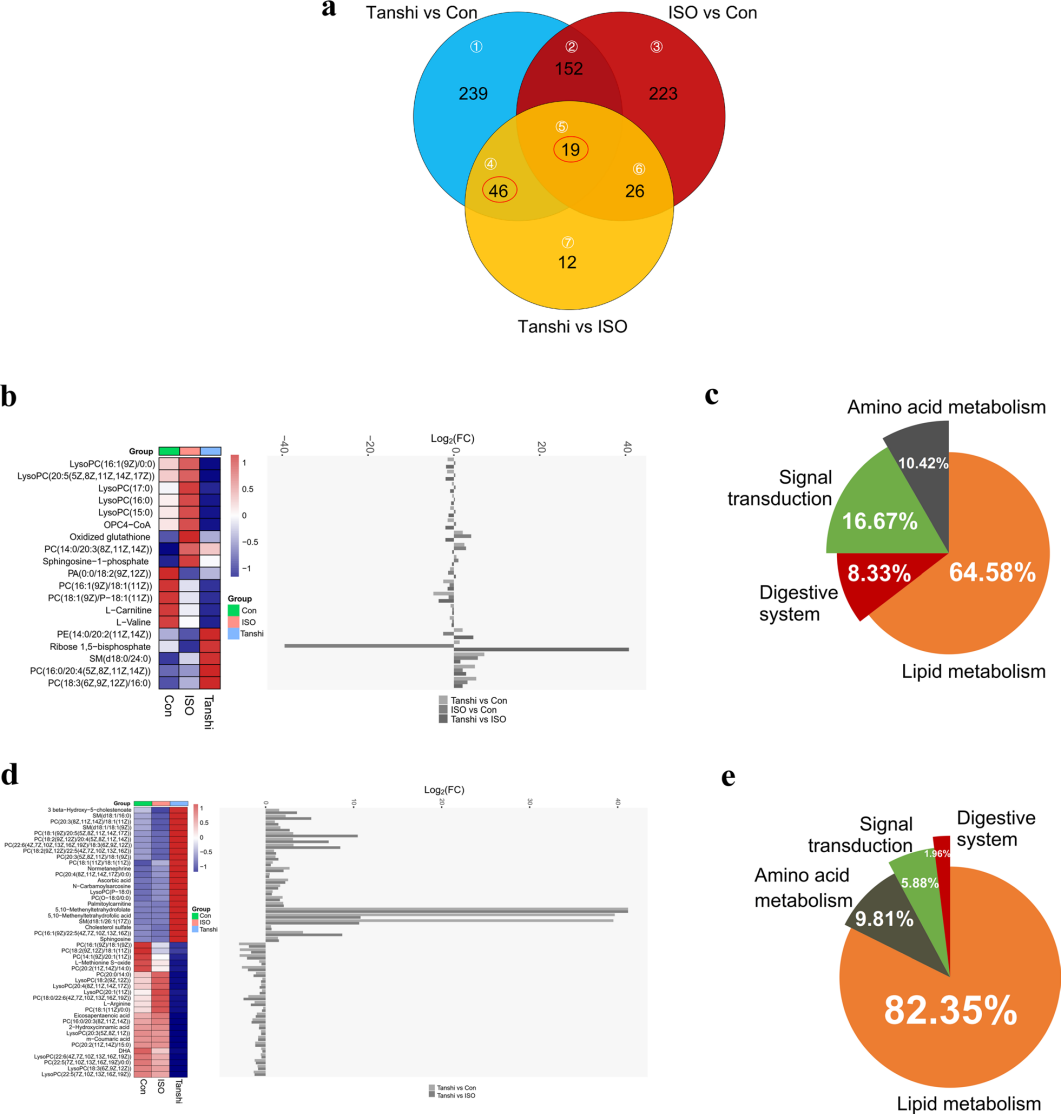
Metabolism disorder contributes to the promotion of myocardial injury in Tanshi mice. **a** Venn analysis. **b** Heatmap of shared differential metabolites in all three comparison groups. **c** KEGG subclass of shared differential metabolites in all three comparison groups. **d** Heatmap of shared differential metabolites in Tanshi versus ISO group and Tanshi versus Con group. **e** KEGG subclass of shared differential metabolites in Tanshi versus ISO group and Tanshi versus Con group

To further reveal the metabolism disorder contributing to the promotion of cardiac injury in ISO-induced myocardial ischemia mice with high temperature high humidity and high-fat diet (also named Tanshi mice), 103 differential metabolites related to the most perturbed metabolisms compared with ISO mice were selected and details of these metabolites were shown in Additional file 4: Table S3. With venn analysis, 19 of these 103 metabolites were found to be changed in all three comparison groups (area ⑤ in Fig. 5a), mainly including Lyso-PCs, PCs, sphingosine-1-phosphate (S1P), OPC4-CoA, L-Carnitine and L-Valine. The relative concentrations of the 19 metabolites altered in all three groups were showed in the heatmap (Fig. 5b), and as shown in Fig. 5c, metabolic disturbances were mainly associated with Lipid metabolism (64.58%), Signal transduction (16.67%), Amino acid metabolism (10.42%) and Digestive system (8.33%). In addition, 46 of these 103 metabolites were found to be changed both in Tanshi versus ISO group and Tanshi versus Con group (area ④ in Fig. 5a), mainly including Lyso-PCs, PCs, SMs, 3 beta-Hydroxy-5-cholestenoate, Normetanephrine, Ascorbic acid, N-Carbamoylsarcosine, Palmitoylcarnitine, 5,10-Methenyltetrahydrofolate, Cholesterol sulfate, Sphingosine, L-Methionine S-oxide, L-Arginine, Eicosapentaenoic acid, 2-Hydroxycinnamic acid, m-Coumaric acid and DHA. The relative concentrations of the 46 metabolites altered in all three groups were showed in the heatmap (Fig. 5d), and as shown in Fig. 5e, metabolic disturbances were mainly associated with Lipid metabolism (82.35%), Amino acid metabolism (9.81%), Signal transduction (5.88%), and Digestive system (1.96%).

## Discussion

ISO-induced myocardial injury animal models are characterized by myocardial hypertrophy, ischemia and even heart failure, which contributes effectively to understanding of the cellular alterations as well as the pathological changes in the heart [10]. Compare with those animal models induced by surgical procedures (e.g., coronary artery ligation, aorta banding), ISO administration provided a simple and non-invasive method for inducing myocardial injury with lower mortality and higher reproducibility. In our present study, a myocardial injury mouse model was introduced by ISO administration combined with high temperature and high humidity and high fat diet to simulate the TCM syndrome of “phlegm-damp”. The results showed the levels of TC, TG and LDL-C in serum of Tanshi mice were significantly higher, and the values of IVS and LVPW were worse in Tanshi mice, which were fed with western diet and housed in room temperature of 35 ± 0.5 °C and relative humidity of 90 ± 5%, compared with the ISO mice. Moreover, mice in Tanshi group developed a significantly increase in cardiac index compared with ISO mice. Significantly more server myocardial fibrosis was also found in Tanshi mice compared with the ISO mice. All these results suggested that high-fat diet with high temperature and high humidity promotes in ISO-induced myocardial injury in mice.

Disorders in cardiac metabolism involved in the pathogenesis of most cardiovascular diseases since diverse sources such as carbohydrates, lipids, lactate, amino acids are needed for supply of energy [11]. Moreover, disturbances in cardiac metabolism may affect systemic metabolism initiating a vicious cycle that accelerates the development of cardiovascular diseases. Metabolomics is downstream of transcriptional, translational, and posttranslational processes, and considered to be the most sensitive to environmental and dietary influences, including such as dietary intake, gut microbiota variation, physical activity [12]. Circulating metabolites reflect immediate information of physiologic condition, and findings from metabolomics studies potentially offer key insights into cardiovascular diseases pathogenesis [13, 14]. Accumulating experimental and clinical studies of metabolomics in cardiovascular diseases have been reported and the applications of which have been well summarized [15, 16]. For instance, in vivo experiments on cardiac hypertrophy and heart failure using transverse aortic constriction (TAC) and myocardial infarction (MI) in mice revealed increased acylcarnitines and decreased tricarboxylic acid (TCA) cycle intermediates in heart tissue [17], accumulation of branched-chain amino acids (BCAA) and branched-chain α-ketoacids (BCKA) in heart tissue and plasma [18, 19], decreased cardiac fatty acid oxidation and increased ketone oxidation in heart tissue [20, 21]. Two early clinical studies of patients with coronary artery disease (CAD) by using Targeted LC–MS showed that higher levels of BCAAs and urea cycle metabolites were associated with CAD [22, 23]. Other clinical studies with patients undergoing elective cardiac catheterization respectively revealed that higher levels of trimethylamine-*N*-oxide (TMAO) were associated with CVD [24, 25], and long-chain dicarboxylacylcarnitines, BCAAs, and fatty acids were associated with death or MI [26].

Metabolomic profiling of ISO-induced myocardial infarction has also been revealed in animal studies. By using the ultra-performance liquid chromatography/time-of-flight mass spectrometry (UPLC/TOF–MS), 13 lipid biomarkers (e.g. Lyso-PCs and fatty acids) had been identified in serum of ISO-induced MI rats [27]. An integrated UPLC-Q/TOF–MS and ^1^H NMR multiplatform based on a tissue-targeted metabonomic approach revealed 22 metabolites (14 down-regulated and 8 up-regulated) as potential biomarkers and 5 pathways (e.g. taurine and hypotaurine metabolism) as the most relevant pathways in ISO-induced MI rats [28].

In our present study, PCA, PLS-DA and heatmaps of differential metabolites indicated clear differentiations among control mice, ISO mice and Tanshi mice, which were stimulated by ISO injection with high temperature and high humidity and high-fat diet. Our study also confirmed the presence of specific metabolites and disturbed metabolic pathways by comparing ISO mice and Tanshi mice. Our data suggested that impaired lipid metabolism (mainly glycerophospholipid metabolism), amino acid metabolism (mainly arginine–proline metabolism), digestive system (mainly protein digestion and absorption) and signal transduction (mainly sphingolipid signaling pathway) are associated with the promotion of ISO-induced myocardial injury in mice with high temperature and high humidity and high-fat diet. The lysophosphatidylcholines (lysoPCs), phosphatidylcholines (PCs), sphingomyelins (SMs) were the main groups of metabolites that showed a difference between ISO and Tanshi mice, which play deleterious roles in pathogenesis of ISO-induced myocardial injury.

The major classes of glycerophospholipid include phosphatidylcholine, phosphatidylethanolamine, phosphatidylserine, phosphatidylinositol, and phosphatidic acid [29]. Phosphatidylcholines (PC), which constitutes approximately 40% of human cardiomyocytes, have received special attention in cardiovascular research. The activation of PC generation may lead to the accumulation of SM and Ceramide (Cer) which promote cell apoptosis. Increased PC in patients with angina pectoris and myocardial infarction had been demonstrated in previous clinical studies [29, 30]. According to our results, two PCs were found higher (PC(16:0/20:4(5Z,8Z,11Z,14Z)), PC(18:3(6Z,9Z,12Z)/16:0), while three PCs were lower (PC(14:0/20:3(8Z,11Z,14Z)), PC(16:1(9Z)/18:1(11Z)), PC(18:1(9Z)/P-18:1(11Z))) in all three comparison groups. In addition, ten PCs were found higher in Tanshi mice compared with ISO and control mice (as shown in Fig. 4b). On the other hand, we found five decreased LysoPC (LysoPC(16:1(9Z)/0:0), LysoPC(20:5(5Z,8Z,11Z,14Z,17Z)), LysoPC(17:0), LysoPC(16:0), LysoPC(15:0)) in all three comparison groups and seven decreased LysoPC (LysoPC(18:2(9Z,12Z)), LysoPC(20:4(8Z,11Z,14Z,17Z)), LysoPC(20:1(11Z)), LysoPC(20:3(5Z,8Z,11Z)), LysoPC(22:6(4Z,7Z,10Z,13Z,16Z,19Z)), LysoPC(18:3(6Z,9Z,12Z)), LysoPC(22:5(7Z,10Z,13Z,16Z,19Z))) in Tanshi mice compared with ISO and control mice. LysoPC has proatherogenic roles in myocardial infarction by inducing monocyte recruitment, macrophage and smooth muscle cell proliferation, endothelial adhesion molecules expression and endothelial dysfunction. LysoPC may also participate in the oxidative stress process through the oxidase pathway and the activation of protein kinase C in blood vessels. The reduction of Lyso-PCs in Tanshi mice may be due to the activated Phospholipase A2 (PLA2) during the breakdown of membrane, which leads to the decreased generation of lysoPCs. The increased PC and decreased LysoPC showed in our study indicated an underlying lipid disturbance in ISO-induced mice model with high temperature and high humidity and high-fat diet that may be associated with the promotion of myocardial injury.

Sphingolipids, components of all eukaryotic cell membranes, have been shown to be related to regulation of various biological processes such as cell proliferation, migration and differentiation, which contribute to the pathogenesis of diverse diseases including cardiovascular diseases, neurodegenerative diseases and cancer [31]. Sphingosine, the intermediate lipid between Ceramide (Cer) and S1P, can be converted to S1P by the action of sphingosine kinases (SphK), while Cer can be converted to sphingosine by the action of ceramidases [32]. The balance between Cer and S1P is a crucial determinant of cellular responses to cytokines, inflammation and oxidative stress Increases in SM and sphingosine promote cell death such as apoptosis, whereas increases in S1P is beneficial to cell survival and proliferation [33]. Sphingosine is also a signal transduction factor of TNF-α during myocardial injury, which reduced myocardial contractility, inhibited the transport of calcium ions in cardiomyocytes, and induced calcium overload [34]. It was found in our present study that the levels of SM(d18:0/16:0), SM(d18:0/24:0), SM(d18:1/26:1(17Z)) and sphingosine were higher, while the level of S1P was lower in Tanshi mice compared with ISO and Con mice. These results indicated that the increase oxidative stress, calcium overload and cell death caused by alterations of all these sphingolipids might be the explanation for the promotion of ISO-induced myocardial injury in mice with high temperature and high humidity and high-fat diet.

Additionally, our study also showed that arginine–proline metabolism is one of the most relevant pathways in Tanshi versus ISO group. L-Arginine, a semiessential amino acid, is the substrate for nitric oxide synthase (NOS), which play a crucial role in regulation of nitric oxide (NO) generation [35]. The decline of L-Arginine and disorder of its metabolism may cause dysfunction of endothelial NOS (eNOS), leading to decreased NO generation, which results in a sequent injury to vessels and organs including the heart. The activation of inducible NOS (iNOS), which leads to decrease of arginine, has been reported in ISO-induced myocardial apoptosis and injury by increasing reactive oxygen species (ROS), and arginine pretreatment can attenuate ISO-induced cardiac hypertrophy via regulating the expression of iNOS and eNOS [36, 37]. In our study, a significant decrease of L-Arginine was found in Tanshi mice compare with ISO and Con mice, which might be the result of the underlying mechanism (e.g., eNOS dysfunction, cell apoptosis and oxidative stress) in the promotion of ISO-induced myocardial injury in mice with high temperature and high humidity and high-fat diet.

## Conclusions

In conclusion, our current study provides new insights into contribution of metabolism disorder in promotion of ISO-induced myocardial injury in mice with high temperature and high humidity and high-fat diet. Furthermore, our data may provide new targets for clinical diagnosis and pharmacologic treatment of cardiovascular disease with syndrome of “phlegm-damp”. Nonetheless, there are some limitations in our present study. For example, the differences between the effects of high-fat diet, separately from the high temperature and the high humidity are still unknown. In addition, ApoE−/− mice were used in the present study due to their advantages in simulating hyperlipidemia and atherosclerosis, which are two of the most common characteristics of “phlegm-damp” syndrome in patients with CVDs. However, given significant phenotypic differences between ApoE+/+ mice and ApoE−/− mice were observed in their lipid and lipoprotein profiles, the cholesterol and metabolomic differences found in Tanshi mice (ApoE−/− mice with ISO injection and environmental challenges) of our current study could potentially be attributed to the ApoE−/− phenotype and the independent effects of temperature, humidity and high-fat diet remain unknown. Therefore, in order to improve the validity of our conclusion that metabolism disorders, especially lipid metabolism disorder, contribute to the mechanisms underlying phlegm-damp syndrome, the differences between ApoE−/− mice and ApoE+/+ mice under specific environmental conditions (high temperature/high humidity/high-fat diet) as well as the differences between ApoE−/− mice with and without environmental challenges are needed to be further studied.

## Supplementary Information

**Additional file 1. Fig S1 Body weight.** The body weights of mice in control, ISO and Tanshi groups were measured every two weeks.

**Additional file 2. Table S1.** The top 20 up-regulated differential metabolites between Tanshi group and ISO group.

**Additional file 3. Table S2.** The top 20 down-regulated differential metabolites between Tanshi group and ISO group. 

**Additional file 4. Table S3.** The 103 differential metabolites related to the most perturbed metabolisms in Tanshi group compared with ISO mice.

## Data Availability

The datasets used and/or analysed during the current study are available in the metabolights repository, https://www.ebi.ac.uk/metabolights/MTBLS3886/descriptors.

## Abbreviations

BCAA: Branched-chain amino acids
BCKA: Branched-chain α-ketoacids
CAD: Coronary artery disease
Cer: Ceramide
CVDs: Cardiovascular diseases
eNOS: Endothelial NOS
iNOS: Inducible NOS
ISO: Isoproterenol
IVS: Inter ventricular septal thickness
LVPW: Left ventricular posterior wall thickness
LysoPC: Lysophosphatidylcholine
MI: Myocardial infarction
NO: Nitric oxide
NOS: Nitric oxide synthase
PC: Phosphatidylcholine
PCA: Principle component analysis
PLA2: Phospholipase A2
PLS-DA: Partial least squares discriminant analysis
ROS: Reactive oxygen species
SM: Sphingomyelin
TAC: Transverse aortic constriction
SphK: Sphingosine kinases
TCA: Tricarboxylic acid
TCM: Traditional Chinese Medicine
TMAO: Trimethylamine-*N*-oxide
VIP: Variable influence on projection
