# Supplementary material for: Metabolism disorder promotes isoproterenol-induced myocardial injury in mice with high temperature and high humidity and high-fat diet
Source: BMC Cardiovasc Disord. 2022 Mar 30;22:133. doi: 10.1186/s12872-022-02583-z (PMC8966251; doi:10.1186/s12872-022-02583-z)
Supplement: Supplementary file 1 — Additional file 1. Fig S1 Body weight. The body weights of mice in control, ISO and Tanshi groups were measured every two weeks. [file 12872_2022_2583_MOESM1_ESM.docx]

**Additional file 1**


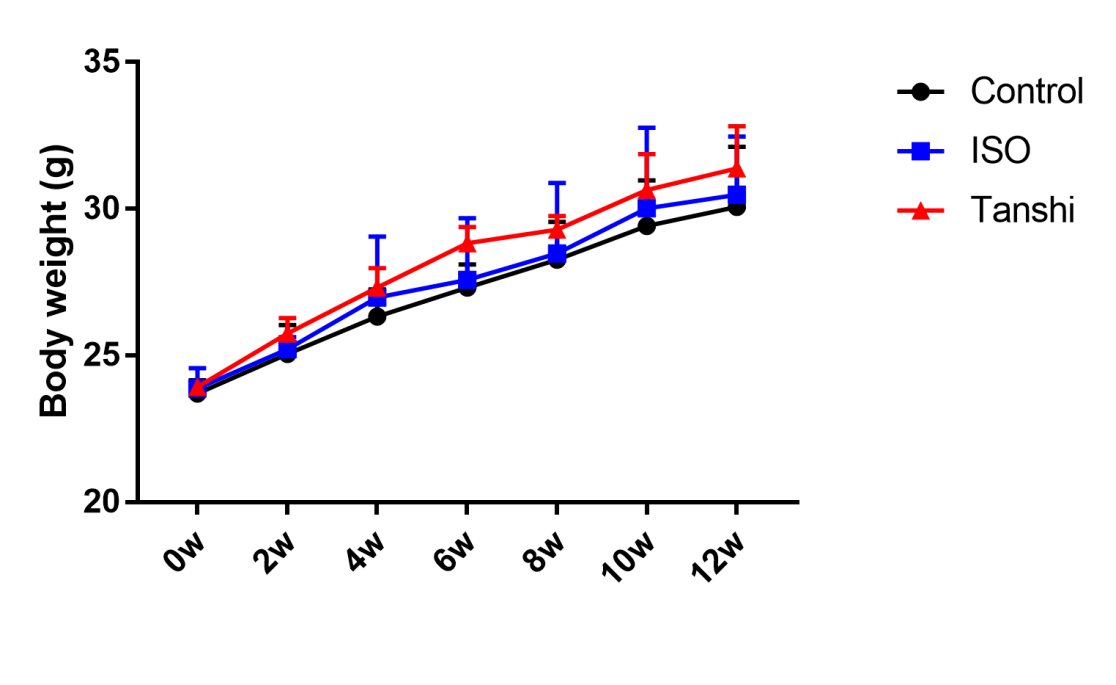


**Figure S1 Body weight**. The body weights of mice in control, ISO and Tanshi groups were measured every two weeks.
