# Supplementary material for: Metabolism disorder promotes isoproterenol-induced myocardial injury in mice with high temperature and high humidity and high-fat diet
Source: BMC Cardiovasc Disord. 2022 Mar 30;22:133. doi: 10.1186/s12872-022-02583-z (PMC8966251; doi:10.1186/s12872-022-02583-z)
Supplement: Supplementary file 2 — Additional file 2. Table S1. The top 20 up-regulated differential metabolites between Tanshi group and ISO group. [file 12872_2022_2583_MOESM2_ESM.docx]

**Additional file 2**

**Table S1** The top 20 up-regulated differential metabolites between Tanshi group and ISO group

| Metabolites | Super Class | Class | VIP | adj.P-value | | log2(FC) |
| --- | --- | --- | --- | --- | --- | --- |
| Isoricinoleic Acid  3 beta-Hydroxy-5-cholestenoate  12(13)-EpOME  xi-5-Hydroxydodecanoic acid  Phytyl phosphate  (6RS,24R)-24,25-dihydroxyvitamin D3 6,19-sulfur dioxide adduct  Niacinamide  Diphenylphosphine isopropyl ester  SM(d18:0/16:0)  7-Mercaptoheptanoic acid  PC(O-18:0/0:0)  1,14-Pentadecadien-3-one  MG(0:0/20:5(5Z,8Z,11Z,14Z,17Z)/0:0)  Trichloroethanol glucuronide  Lamivudine-triphosphate  1alpha,25-dihydroxy-2alpha-hydroxymethyl-19-norvitamin D3  4-(2,6,6-Trimethyl-1-cyclohexenyl)-2-butanol  Stigmatellin Y  Terbutaline-1-glucuronide  Sphingosine | Lipids and lipid-like molecules  Lipids and lipid-like molecules  Lipids and lipid-like molecules  Lipids and lipid-like molecules  Unclassified  Unclassified  Organoheterocyclic compounds  Unclassified  Lipids and lipid-like molecules  Unclassified  Lipids and lipid-like molecules  Lipids and lipid-like molecules  Lipids and lipid-like molecules  Organic oxygen compounds  Nucleosides, nucleotides, and analogues  Lipids and lipid-like molecules  Lipids and lipid-like molecules  Unclassified  Unclassified  Lipids and lipid-like molecules | Fatty Acyls  Steroids and steroid derivatives  Fatty Acyls  Fatty Acyls  Unclassified  Unclassified  Pyridines and derivatives  Unclassified  Sphingolipids  Unclassified  Glycerophospholipids  Fatty Acyls  Glycerolipids  Organooxygen compounds  Nucleoside and nucleotide analogues  Sterol Lipids  Prenol lipids  Unclassified  Unclassified  Sphingolipids | 1.088418179  1.092883904  1.103460557  1.362662748  1.562208801  1.234560779  3.539697203  3.230409086  4.068425766  3.359504986  1.627070591  1.420750559  1.901782019  29.38290512  1.102862564  1.276224132  3.373531256  1.36121943  25.78062349  1.00246704 | | 1.38109E-07  2.38584E-06  2.79656E-06  5.8758E-06  5.94396E-06  6.00013E-06  7.38904E-06  7.92159E-06  1.23772E-05  1.51963E-05  1.66469E-05  1.77408E-05  2.14041E-05  2.22694E-05  2.26007E-05  2.38398E-05  3.04886E-05  3.30556E-05  3.64102E-05  3.8197E-05 | 2.54933394  3.560261482  2.068670378  2.469305755  3.301047974  39.69495978  1.670538931  14.30077823  9.428304479  42.60352531  1.622512081  3.075391051  5.87697341  15.05028736  39.41007243  2.32713009  3.363889561  4.009251151  7.733949728  1.506840747 |
