# Supplementary material for: Metabolism disorder promotes isoproterenol-induced myocardial injury in mice with high temperature and high humidity and high-fat diet
Source: BMC Cardiovasc Disord. 2022 Mar 30;22:133. doi: 10.1186/s12872-022-02583-z (PMC8966251; doi:10.1186/s12872-022-02583-z)
Supplement: Supplementary file 3 — Additional file 3. Table S2. The top 20 down-regulated differential metabolites between Tanshi group and ISO group. [file 12872_2022_2583_MOESM3_ESM.docx]

**Additional file 3**

**Table 2** The top 20 down-regulated differential metabolites between Tanshi group and ISO group

| Metabolites | Super Class | Class | VIP | adj.P-value | | log2(FC) |
| --- | --- | --- | --- | --- | --- | --- |
| 26,26,26,27,27,27-hexafluoro-25-hydroxyvitamin D2  LysoPE(0:0/20:5(5Z,8Z,11Z,14Z,17Z))  Haloxyfop  OPC4-CoA  LysoPE(20:5(5Z,8Z,11Z,14Z,17Z)/0:0)  LysoPC(16:1(9Z)/0:0)  PC(15:1(9Z)/0:0)  Norophthalmic acid  Indoleacrylic acid  LysoPE(16:1(9Z)/0:0)  PE(16:1(9Z)/0:0)  PIM1(18:0/16:2(9Z,12Z))  PS(19:1(9Z)/22:2(13Z,16Z))  4-formyl Indole  Tryptophanol  Pyridine N-oxide glucuronide  PE(22:1(11Z)/0:0)  [4-(7-hydroxy-3,4-dihydro-2H-1-benzopyran-3-yl)phenyl]oxidanesulfonic acid  4-Ethynylaniline  L-Tryptophan | Lipids and lipid-like molecules  Lipids and lipid-like molecules  Unclassified  Lipids and lipid-like molecules  Lipids and lipid-like molecules  Lipids and lipid-like molecules  Lipids and lipid-like molecules  Organic acids and derivatives  Organoheterocyclic compounds  Lipids and lipid-like molecules  Lipids and lipid-like molecules  Lipids and lipid-like molecules  Unclassified  Unclassified  Organoheterocyclic compounds  Organic oxygen compounds  Lipids and lipid-like molecules  Phenylpropanoids and polyketides  Unclassified  Organoheterocyclic compounds | Sterol Lipids  Glycerophospholipids  Unclassified  Fatty Acyls  Glycerophospholipids  Glycerophospholipids  Glycerophospholipids  Carboxylic acids and derivatives  Indoles and derivatives  Glycerophospholipids  Glycerophospholipids  Glycerophospholipids  Unclassified  Unclassified  Indoles and derivatives  Organooxygen compounds  Glycerophospholipids  Isoflavonoids  Unclassified  Indoles and derivatives | 1.091961624  4.929989849  1.405644862  6.096397235  1.597285837  16.75063795  1.35377606  1.94088503  16.87638941  1.602917372  1.389535866  1.465783273  1.355939817  1.789526933  1.233555758  1.075263032  1.417205308  7.34137951  1.02395811  2.114766241 | | 8.68221E-08  9.33073E-08  1.08764E-07  1.83675E-07  9.62857E-07  1.39959E-06  2.90078E-06  2.90078E-06  3.00103E-06  3.04996E-06  3.05969E-06  3.87723E-06  3.95378E-06  4.80501E-06  5.36057E-06  5.51069E-06  5.72115E-06  6.51683E-06  6.75322E-06  6.84676E-06 | -2.967438423  -2.747432213  -4.478342917  -1.975383947  -2.231308141  -1.834147821  -1.811810094  -5.853816439  -2.205448311  -1.660100017  -1.768080671  -2.081104767  -2.258597125  -2.109123085  -2.240859656  -1.620640964  -1.52323455  -3.859043479  -1.590396832  -3.195371749 |
