# Supplementary material for: Metabolism disorder promotes isoproterenol-induced myocardial injury in mice with high temperature and high humidity and high-fat diet
Source: BMC Cardiovasc Disord. 2022 Mar 30;22:133. doi: 10.1186/s12872-022-02583-z (PMC8966251; doi:10.1186/s12872-022-02583-z)
Supplement: Supplementary file 4 — Additional file 4. Table S3. The 103 differential metabolites related to the most perturbed metabolisms in Tanshi group compared with ISO mice. [file 12872_2022_2583_MOESM4_ESM.docx]

**Additional file 4**

**Table S3**

The 103 differential metabolites related to the most perturbed metabolisms in Tanshi group compared with ISO mice

| Metabolites | Annotation | Sub Class | VIP | P-value | log2(FC) |
| --- | --- | --- | --- | --- | --- |
| OPC4-CoA | alpha-Linolenic acid metabolism | Lipid metabolism | 6.096397 | 4.39E-10 | -1.97538 |
| LysoPC(16:1(9Z)/0:0) | Choline metabolism in cancer \|Glycerophospholipid metabolism | Cancers: Overview \|Lipid metabolism | 16.75064 | 6.7E-09 | -1.83415 |
| 3 beta-Hydroxy-5-cholestenoate | Primary bile acid biosynthesis | Lipid metabolism | 1.092884 | 1.62E-08 | 3.560261 |
| 12(13)-EpOME | Linoleic acid metabolism | Lipid metabolism | 1.103461 | 2.08E-08 | 2.06867 |
| Tryptophanol | Tryptophan metabolism | Amino acid metabolism | 1.233556 | 5.74E-08 | -2.24086 |
| L-Tryptophan | Central carbon metabolism in cancer \|Protein digestion and absorption\| Biosynthesis of amino acids \|Aminoacyl-tRNA biosynthesis \|Mineral absorption \|2-Oxocarboxylic acid metabolism \|African trypanosomiasis \|Serotonergicsynapse \|Glycine, serine and threonine metabolis \|Tryptophan metabolism \|Phenylalanine, tyrosine and tryptophan biosynthesis | Cancers: Overview \|Digestive system \|Global and overview maps \|Translation \|Digestive system \|Global and overview maps \|Infectious diseases: Parasitic \|Nervous system \|Amino acid metabolism \|Amino acid metabolism \|Amino acid metabolism | 2.114766 | 9.29E-08 | -3.19537 |
| Niacinamide | Vitamin digestion and absorption \|Nicotinate and nicotinamide metabolism | Digestive system \|Metabolism of cofactors and vitamins | 3.539697 | 1.04E-07 | 1.670539 |
| LysoPC(15:0) | Choline metabolism in cancer \|Glycerophospholipid metabolism | Cancers: Overview \|Lipid metabolism | 5.195507 | 1.37E-07 | -1.24263 |
| SM(d18:0/16:0) | Sphingolipid signaling pathway \|Sphingolipid metabolism \|Necroptosis | Signal transduction \|Lipid metabolism \|Cell growth and death | 4.068426 | 2.32E-07 | 9.428304 |
| LysoPC(20:5(5Z,8Z,11Z,14Z,17Z)) | Choline metabolism in cancer \|Glycerophospholipid metabolism | Cancers: Overview \|Lipid metabolism | 12.61956 | 3.31E-07 | -1.93538 |
| PC(O-18:0/0:0) | Ether lipid metabolism | Lipid metabolism | 1.627071 | 3.56E-07 | 1.622512 |
| Sphingosine | Sphingolipid signaling pathway \|Sphingolipidmetabolism \|Necroptosis \|Apoptosis | Signal transduction \|Lipid metabolism \|Cell growth and death \|Cell growth and deat | 1.002467 | 1.31E-06 | 1.506841 |
| TG(16:0/16:0/18:0) | Vitamin digestion and absorption \|Fat digestion and absorption \|Regulation of lipolysis in adipocytes \|Thermogenesis \|Glycerolipidmetabolism \|Cholesterol metabolism \|Insulin resistance | Digestive system \|Digestive system \|Endocrine system \|Environmental adaptation \|Lipid metabolism \|Digestive system \|Endocrine and metabolic diseases | 1.194602 | 1.33E-06 | -2.08056 |
| LysoPC(16:0) | Choline metabolism in cancer \|Glycerophospholipid metabolism | Cancers: Overview \|Lipid metabolism | 15.44508 | 1.44E-06 | -0.64172 |
| PE(14:0/20:2(11Z,14Z)) | Glycerophospholipid metabolism \|Retrograde endocannabinoid signaling \|Glycosylphosphatidylinositol (GPI)-anchor biosynthesis \|Autophagy – other \|Autophagy – animal \|Kaposi sarcoma-associated herpesvirus infection | Lipid metabolism \|Nervous system \|Glycan biosynthesis and metabolism \|Transport and catabolism \|Transport and catabolism \|Infectious diseases: Viral | 1.79225 | 1.78E-06 | 4.533692 |
| LysoPC(17:0) | Choline metabolism in cancer \|Glycerophospholipid metabolism | Cancers: Overview \|Lipid metabolism | 4.387772 | 1.9E-06 | -0.87066 |
| SM(d18:1/18:1(9Z)) | Sphingolipid signaling pathway \|Sphingolipid metabolism \|Necroptosis | Signal transduction \|Lipid metabolism \|Cell growth and death | 4.027831 | 3.86E-06 | 2.730755 |
| Ribose 1,5-bisphosphate | Pentose phosphate pathway \|Phosphonate and phosphinate metabolism | Carbohydrate metabolism\|Metabolism of other amino acids | 1.771129 | 4.87E-06 | 40.79642 |
| 5,10-Methenyltetrahydrofolic acid | One carbon pool by folate \|Carbon metabolism | Metabolism of cofactors and vitamins \|Global and overview maps | 1.203077 | 5.39E-06 | 10.78407 |
| PC(16:1(9Z)/22:5(4Z,7Z,10Z,13Z,16Z)) | Choline metabolism in cancer \|Glycerophospholipid metabolism \|Linoleic acid metabolism \|Retrograde endocannabinoid signaling \|alpha-Linolenic acid metabolism \|Arachidonic acid metabolism | Cancers: Overview \|Lipid metabolism \|Lipid metabolism \|Nervous system\| Lipid metabolism \|Lipid metabolism | 8.589377 | 2.21E-05 | 8.706531 |
| Palmitoylcarnitine | Fatty acid degradation \|Fatty acid metabolism | Lipid metabolism \|Global and overview maps | 7.27105 | 2.25E-05 | 2.066143 |
| PE(20:3(8Z,11Z,14Z)/P-16:0) | Glycerophospholipid metabolism \|Retrograde endocannabinoid signaling \|Glycosylphosphatidylinositol (GPI)-anchor biosynthesis \|Autophagy - other \|Autophagy – animal \|Kaposi sarcoma-associated herpesvirus infection | Lipid metabolism \|Nervous system \|Glycan biosynthesis and metabolism \|Transport and catabolism \|Transport and catabolism \|Infectious diseases: Viral | 2.947095 | 2.69E-05 | 3.652725 |
| LysoPC(18:3(6Z,9Z,12Z)) | Choline metabolism in cancer \|Glycerophospholipid metabolism | Cancers: Overview \|Lipid metabolism | 4.422412 | 3.52E-05 | -0.85991 |
| PC(20:4(5Z,8Z,11Z,14Z)/16:0) | Choline metabolism in cancer \|Glycerophospholipid metabolism \|Linoleic acid metabolism \|Retrograde endocannabinoid signaling \|alpha-Linolenic acid metabolism \|Arachidonic acid metabolism | Cancers: Overview \|Lipid metabolism \|Lipid metabolism \|Nervous system\| Lipid metabolism \|Lipid metabolism | 5.039235 | 6.25E-05 | 4.661323 |
| PE(20:5(5Z,8Z,11Z,14Z,17Z)/P-18:1(9Z)) | Glycerophospholipid metabolism \|Retrograde endocannabinoid signaling \|Glycosylphosphatidylinositol (GPI)-anchor biosynthesis \|Autophagy – other \|Autophagy – animal \|Kaposi sarcoma-associated herpesvirus infection | Lipid metabolism \|Nervous system \|Glycan biosynthesis and metabolism \|Transport and catabolism \|Transport and catabolism \|Infectious diseases: Viral | 1.198323 | 6.9E-05 | 39.72171 |
| PC(15:0/15:0) | Choline metabolism in cancer \|Glycerophospholipid metabolism \|Linoleic acid metabolism \|Retrograde endocannabinoid signaling \|alpha-Linolenic acid metabolism \|Arachidonic acid metabolism | Cancers: Overview \|Lipid metabolism \|Lipid metabolism \|Nervous system\| Lipid metabolism \|Lipid metabolism | 1.761743 | 8.1E-05 | 9.451301 |
| LysoPC(18:2(9Z,12Z)) | Choline metabolism in cancer \|Glycerophospholipid metabolism | Cancers: Overview \|Lipid metabolism | 11.92463 | 8.3E-05 | -0.52344 |
| PC(18:2(9Z,12Z)/20:4(5Z,8Z,11Z,14Z)) | Choline metabolism in cancer \|Glycerophospholipidmetabolism \|Linoleic acid metabolism \|Retrograde endocannabinoid signaling \|alpha-Linolenic acid metabolism \|Arachidonic acid metabolism | Cancers: Overview \|Lipid metabolism \|Lipid metabolism \|Nervous system\| Lipid metabolism \|Lipid metabolism | 5.82681 | 8.72E-05 | 7.162221 |
| PE(20:3(8Z,11Z,14Z)/15:0) | Glycerophospholipid metabolism \|Retrograde endocannabinoid signaling \|Glycosylphosphatidylinositol (GPI)-anchor biosynthesis \|Autophagy – other \|Autophagy – animal \|Kaposi sarcoma-associated herpesvirus infection | Lipid metabolism \|Nervous system \|Glycan biosynthesis and metabolism \|Transport and catabolism \|Transport and catabolism \|Infectious diseases: Viral | 1.133272 | 9.21E-05 | 39.56872 |
| L-Arginine | Central carbon metabolism in cancer \|Protein digestion and absorption \|Biosynthesis of amino acids \|Aminoacyl-tRNA biosynthesis \|ABC transporters\| Arginine and proline metabolism \|mTOR signaling pathway \|Salmonella infection \|Chagas disease (American trypanosomiasis) \|D-Arginine and D-ornithine metabolism \|Amyotrophic lateral sclerosis (ALS) \|Amoebiasis \|Arginine biosynthesis | Cancers: Overview \|Digestive system \|Global and overview maps \|Translation \|Membrane transport \|Amino acid metabolism \|Signal transduction \|Infectious diseases: Bacterial \|Infectious diseases: Parasitic \|Metabolism of other amino acids \|Neurodegenerative diseases \|Infectious diseases: Parasitic \|Amino acid metabolism | 3.718508 | 0.000111 | -1.68295 |
| gamma-Glutamylalanine | Glutathione metabolism | Metabolism of other amino acids | 1.218415 | 0.000135 | -2.645 |
| PC(18:3(6Z,9Z,12Z)/16:0) | Choline metabolism in cancer \|Glycerophospholipid metabolism \|Linoleic acid metabolism \|Retrograde endocannabinoid signaling \|alpha-Linolenic acid metabolism \|Arachidonic acid metabolism | Cancers: Overview \|Lipid metabolism \|Lipid metabolism \|Nervous system\| Lipid metabolism \|Lipid metabolism | 1.788664 | 0.000135 | 2.036004 |
| SM(d18:1/16:0) | Sphingolipid signaling pathway \|Sphingolipid metabolism \|Necroptosis | Signal transduction \|Lipid metabolism \|Cell growth and death | 1.774389 | 0.000145 | 5.185601 |
| Normetanephrine | Tyrosine metabolism | Amino acid metabolism | 1.018686 | 0.000171 | 2.039739 |
| PC(14:0/22:4(7Z,10Z,13Z,16Z)) | Choline metabolism in cancer \|Glycerophospholipid metabolism \|Linoleic acid metabolism \|Retrograde endocannabinoid signaling \|alpha-Linolenic acid metabolism \|Arachidonic acid metabolism | Cancers: Overview \|Lipid metabolism \|Lipid metabolism \|Nervous system\| Lipid metabolism \|Lipid metabolism | 8.412627 | 0.000183 | 1.931618 |
| LysoPC(22:5(7Z,10Z,13Z,16Z,19Z)) | Choline metabolism in cancer \|Glycerophospholipid metabolism | Cancers: Overview \|Lipid metabolism | 6.901611 | 0.000225 | -1.25946 |
| PC(22:6(4Z,7Z,10Z,13Z,16Z,19Z)/18:3(6Z,9Z,12Z)) | Choline metabolism in cancer \|Glycerophospholipid metabolism \|Linoleic acid metabolism \|Retrograde endocannabinoid signaling \|alpha-Linolenic acid metabolism \|Arachidonic acid metabolism | Cancers: Overview \|Lipid metabolism \|Lipid metabolism \|Nervous system\| Lipid metabolism \|Lipid metabolism | 3.800244 | 0.000249 | 8.480191 |
| Sphingosine-1-phosphate | Sphingolipid signaling pathway \|Fc gamma R-mediated phagocytosis\| Sphingolipidmetabolism \|Phospholipase D signaling pathway\| Apelin signaling pathway \|Tuberculosis \|Calcium signaling pathway \|Neuroactive ligand-receptor interaction | Signal transduction \|Immune system \|Lipid metabolism \|Signal transduction \|Signal transduction \|Infectious diseases: Bacterial \|Signal transduction \|Signaling molecules and interaction | 2.641674 | 0.000282 | -0.45217 |
| PC(22:5(7Z,10Z,13Z,16Z,19Z)/0:0) | Choline metabolism in cancer \|Glycerophospholipid metabolism | Cancers: Overview \|Lipid metabolism | 1.803648 | 0.000302 | -1.1151 |
| LysoPC(20:4(8Z,11Z,14Z,17Z)) | Choline metabolism in cancer \|Glycerophospholipid metabolism | Cancers: Overview \|Lipid metabolism | 6.152703 | 0.000369 | -0.68191 |
| 5,10-Methenyltetrahydrofolate | One carbon pool by folate \|Carbon metabolism | Metabolism of cofactors and vitamins \|Global and overview maps | 1.941687 | 0.00038 | 41.18344 |
| L-Palmitoylcarnitine | Fatty acid degradation \|Fatty acid metabolism | Lipid metabolism \|Global and overview maps | 1.078694 | 0.000403 | 1.260387 |
| Oxidized glutathione | Glutathione metabolism \|Thyroid hormone synthesis \|Ferroptosis | Metabolism of other amino acids \|Endocrine system \|Cell growth and death | 2.677049 | 0.000496 | -1.97204 |
| PE(22:5(7Z,10Z,13Z,16Z,19Z)/20:1(11Z)) | Glycerophospholipid metabolism \|Retrograde endocannabinoid signaling \|Glycosylphosphatidylinositol (GPI)-anchor biosynthesis \|Autophagy – other \|Autophagy – animal \|Kaposi sarcoma-associated herpesvirus infection | Lipid metabolism \|Nervous system \|Glycan biosynthesis and metabolism \|Transport and catabolism \|Transport and catabolism \|Infectious diseases: Viral | 1.162488 | 0.000498 | 1.194008 |
| PE(16:1(9Z)/P-18:0) | Glycerophospholipid metabolism \|Retrograde endocannabinoid signaling \|Glycosylphosphatidylinositol (GPI)-anchor biosynthesis \|Autophagy – other \|Autophagy – animal \|Kaposi sarcoma-associated herpesvirus infection | Lipid metabolism \|Nervous system \|Glycan biosynthesis and metabolism \|Transport and catabolism \|Transport and catabolism \|Infectious diseases: Viral | 1.753605 | 0.000505 | -0.90522 |
| PC(20:4(8Z,11Z,14Z,17Z)/0:0) | Choline metabolism in cancer \|Glycerophospholipid metabolism | Cancers: Overview \|Lipid metabolism | 1.699627 | 0.000615 | 0.409526 |
| N2-Succinyl-L-glutamic acid 5-semialdehyde | Arginine and proline metabolism | Amino acid metabolism | 1.080542 | 0.000666 | -1.33561 |
| TXB2 | Serotonergic synapse \|Bile secretion | Nervous system \|Digestive system | 1.374703 | 0.000699 | -1.23306 |
| PC(18:1(11Z)/0:0) | Choline metabolism in cancer \|Glycerophospholipid metabolism | Cancers: Overview \|Lipid metabolism | 10.44189 | 0.000728 | -0.41848 |
| PC(18:1(9Z)/20:5(5Z,8Z,11Z,14Z,17Z)) | Choline metabolism in cancer \|Glycerophospholipid metabolism \|Linoleic acid metabolism \|Retrograde endocannabinoid signaling \|alpha-Linolenic acid metabolism \|Arachidonic acid metabolism | Cancers: Overview \|Lipid metabolism \|Lipid metabolism \|Nervous system\| Lipid metabolism \|Lipid metabolism | 2.547099 | 0.000868 | 10.46798 |
| PC(16:0/20:4(5Z,8Z,11Z,14Z)) | Choline metabolism in cancer \|Glycerophospholipid metabolism \|Linoleic acid metabolism \|Retrograde endocannabinoid signaling \|alpha-Linolenic acid metabolism \|Arachidonic acid metabolism | Cancers: Overview \|Lipid metabolism \|Lipid metabolism \|Nervous system\| Lipid metabolism \|Lipid metabolism | 4.602179 | 0.000869 | 2.849021 |
| 2-Hydroxycinnamic acid | Phenylalanine metabolism | Amino acid metabolism | 5.820795 | 0.000871 | -0.75492 |
| N-Carbamoylsarcosine | Arginine and proline metabolism | Amino acid metabolism | 1.377482 | 0.000917 | 1.424907 |
| Cholesterol sulfate | Steroid hormone biosynthesis | Lipid metabolism | 1.40694 | 0.001024 | 0.673986 |
| LysoPC(20:1(11Z)) | Choline metabolism in cancer \|Glycerophospholipid metabolism | Cancers: Overview \|Lipid metabolism | 5.167649 | 0.001357 | -0.67321 |
| PC(20:3(8Z,11Z,14Z)/18:1(11Z)) | Choline metabolism in cancer \|Glycerophospholipid metabolism \|Linoleic acid metabolism \|Retrograde endocannabinoid signaling \|alpha-Linolenic acid metabolism \|Arachidonic acid metabolism | Cancers: Overview \|Lipid metabolism \|Lipid metabolism \|Nervous system\| Lipid metabolism \|Lipid metabolism | 6.384163 | 0.001433 | 1.450235 |
| L-Carnitine | Thermogenesis \|Bile secretion | Environmental adaptation \|Digestive system | 2.547059 | 0.001552 | -0.39372 |
| Trolamine | Glycerophospholipid metabolism | Lipid metabolism | 2.540523 | 0.001617 | -4.78203 |
| L-Lysine | Protein digestion and absorption \|Biosynthesis of amino acids \|Aminoacyl-tRNA biosynthesis \|ABC transporters \|Lysine degradation \|Biotin metabolism \|2-Oxocarboxylic acid metabolism | Digestive system \|Global and overview maps \|Translation \|Membrane transport \|Amino acid metabolism \|Metabolism of cofactors and vitamins \|Global and overview maps | 2.359011 | 0.001638 | -0.60331 |
| Ascorbic acid | Vitamin digestion and absorption \|Glutathione metabolism \|Ascorbate and aldarate metabolism \|HIF-1 signaling pathway | Digestive system \|Metabolism of other amino acids \|Carbohydrate metabolism \|Signal transduction | 3.005405 | 0.002039 | 2.235756 |
| LysoPC(P-18:0) | Choline metabolism in cancer \|Glycerophospholipid metabolism | Cancers: Overview \|Lipid metabolism | 3.798094 | 0.002771 | 0.708527 |
| SM(d18:1/26:1(17Z)) | Sphingolipid signaling pathway \|Sphingolipid metabolism \|Necroptosis | Signal transduction \|Lipid metabolism \|Cell growth and death | 1.041399 | 0.003011 | 10.63062 |
| L-Valine | Central carbon metabolism in cancer\| Protein digestion and absorption \|Biosynthesis of amino acids \|Aminoacyl-tRNA biosynthesis \|ABC transporters \|Arginine and proline metabolism \|Mineralabsorption \|Pantothenate and CoA biosynthesis \|2-Oxocarboxylic acid metabolism \|Valine, leucine and isoleucine biosynthesis \|Valine, leucine and isoleucine degradation | Cancers: Overview \|Digestive system \|Global and overview maps \|Translation \|Membrane transport \|Amino acid metabolism \|Digestive system \|Metabolism of cofactors and vitamins \|Global and overview maps \|Amino acid metabolism \|Amino acid metabolism | 2.143821 | 0.003268 | -0.5431 |
| LysoPC(18:1(11Z)) | Choline metabolism in cancer \|Glycerophospholipid metabolism | Cancers: Overview \|Lipid metabolism | 17.32349 | 0.003613 | -0.36771 |
| Pantothenic Acid | Vitamin digestion and absorption \|Pantothenate and CoA biosynthesis \|beta-Alanine metabolism | Digestive system \|Metabolism of cofactors and vitamins \|Metabolism of other amino acids | 1.674675 | 0.003785 | -0.9743 |
| m-Coumaric acid | Phenylalanine metabolism | Amino acid metabolism | 2.89722 | 0.0039 | -0.85468 |
| Eicosapentaenoic acid | Biosynthesis of unsaturated fatty acids | Lipid metabolism | 1.724097 | 0.00425 | -1.13112 |
| PC(15:0/18:2(9Z,12Z)) | Choline metabolism in cancer \|Glycerophospholipid metabolism \|Linoleic acid metabolism \|Retrograde endocannabinoid signaling \|alpha-Linolenic acid metabolism \|Arachidonic acid metabolism | Cancers: Overview \|Lipid metabolism \|Lipid metabolism \|Nervous system\| Lipid metabolism \|Lipid metabolism | 3.688272 | 0.004371 | 1.817102 |
| L-Pipecolic acid | Lysine degradation | Amino acid metabolism | 1.30872 | 0.004631 | -0.419 |
| PC(20:3(8Z,11Z,14Z)/20:4(5Z,8Z,11Z,14Z)) | Choline metabolism in cancer \|Glycerophospholipid metabolism \|Linoleic acid metabolism \|Retrograde endocannabinoid signaling \|alpha-Linolenic acid metabolism \|Arachidonic acid metabolism | Cancers: Overview \|Lipid metabolism \|Lipid metabolism \|Nervous system\| Lipid metabolism \|Lipid metabolism | 2.284799 | 0.004767 | 1.208554 |
| 13R-HODE | Linoleic acid metabolism \|PPAR signaling pathway | Lipid metabolism \|Endocrine system | 1.427567 | 0.004846 | 1.449437 |
| PC(18:2(9Z,12Z)/22:5(4Z,7Z,10Z,13Z,16Z)) | Choline metabolism in cancer \|Glycerophospholipid metabolism \|Linoleic acid metabolism \|Retrograde endocannabinoid signaling \|alpha-Linolenic acid metabolism \|Arachidonic acid metabolism | Cancers: Overview \|Lipid metabolism \|Lipid metabolism \|Nervous system\| Lipid metabolism \|Lipid metabolism | 3.770119 | 0.004902 | 1.186764 |
| LysoPC(18:1(9Z)) | Choline metabolism in cancer \|Glycerophospholipid metabolism | Cancers: Overview \|Lipid metabolism | 6.218618 | 0.005011 | -0.38772 |
| PC(14:0/20:3(8Z,11Z,14Z)) | Choline metabolism in cancer \|Glycerophospholipidmetabolism \|Linoleic acid metabolism \|Retrograde endocannabinoid signaling \|alpha-Linolenic acid metabolism \|Arachidonic acid metabolism | Cancers: Overview \|Lipid metabolism\|Lipid metabolism \|Nervous system\| Lipid metabolism\|Lipid metabolism | 1.831686 | 0.005171 | -0.3867 |
| Creatinine | Arginine and proline metabolism | Amino acid metabolism | 1.263906 | 0.006241 | 1.855679 |
| PC(20:2(11Z,14Z)/15:0) | Choline metabolism in cancer \|Glycerophospholipid metabolism \|Linoleic acid metabolism \|Retrograde endocannabinoid signaling \|alpha-Linolenic acid metabolism \|Arachidonic acid metabolism | Cancers: Overview \|Lipid metabolism\|Lipid metabolism \|Nervous system\| Lipid metabolism\|Lipid metabolism | 1.337017 | 0.006278 | -1.1813 |
| LysoPC(22:6(4Z,7Z,10Z,13Z,16Z,19Z)) | Choline metabolism in cancer \|Glycerophospholipid metabolism | Cancers: Overview \|Lipid metabolism | 12.64919 | 0.007541 | -0.47775 |
| 9R,10S-EpOME | Linoleic acid metabolism | Lipid metabolism | 1.084304 | 0.008088 | 1.635014 |
| LysoPC(20:4(5Z,8Z,11Z,14Z)) | Choline metabolism in cancer \|Glycerophospholipid metabolism | Cancers: Overview \|Lipid metabolism | 11.24463 | 0.008843 | -0.41884 |
| PC(20:2(11Z,14Z)/14:0) | Choline metabolism in cancer \|Glycerophospholipid metabolism \|Linoleic acid metabolism \|Retrograde endocannabinoid signaling \|alpha-Linolenic acid metabolism \|Arachidonic acid metabolism | Cancers: Overview \|Lipid metabolism\|Lipid metabolism \|Nervous system\| Lipid metabolism\|Lipid metabolism | 2.464678 | 0.009049 | -2.01101 |
| DHA | Cysteine and methionine metabolism | Amino acid metabolism | 1.854887 | 0.010456 | -0.37576 |
| PA(0:0/18:2(9Z,12Z)) | Choline metabolism in cancer \|Glycerophospholipid metabolism \|Fc gamma R-mediated phagocytosis \|Phospholipase D signaling pathway \|Fat digestion and absorption \|Pancreaticcancer \|GnRH signaling pathway \|Glycerolipid metabolism \|Phosphatidylinositol signaling system \|Pathways in cancer \|cAMP signaling pathway | Cancers: Overview \|Lipid metabolism \|Immune system \|Signal transduction \|Digestive system \|Cancers: Specific types \|Endocrine system \|Lipid metabolism \|Signal transduction \|Cancers: Overview \|Signal transduction | 1.260642 | 0.012047 | 0.460703 |
| PC(18:1(9Z)/P-18:1(11Z)) | Choline metabolism in cancer \|Glycerophospholipid metabolism \|Linoleic acid metabolism \|Retrograde endocannabinoid signaling \|alpha-Linolenic acid metabolism \|Arachidonic acid metabolism | Cancers: Overview \|Lipid metabolism \|Lipid metabolism \|Nervous system\| Lipid metabolism \|Lipid metabolism | 1.782316 | 0.012799 | -3.55702 |
| Corticosterone | Regulation of lipolysis in adipocytes \|Priondiseases\| Aldosterone synthesis and secretion \|Steroid hormone biosynthesis | Endocrine system \|Neurodegenerative diseases \|Endocrine system \|Lipid metabolism | 1.408667 | 0.013469 | 0.786177 |
| Aminoadipic acid | Biosynthesis of amino acids \|Lysine degradation \|2-Oxocarboxylic acid metabolism | Global and overview maps \|Amino acid metabolism \|Global and overview maps | 1.454666 | 0.013958 | 0.57262 |
| PC(20:3(5Z,8Z,11Z)/18:1(9Z)) | Choline metabolism in cancer \|Glycerophospholipid metabolism \|Linoleic acid metabolism \|Retrograde endocannabinoid signaling \|alpha-Linolenic acid metabolism \|Arachidonic acid metabolism | Cancers: Overview \|Lipid metabolism \|Lipid metabolism \|Nervous system\| Lipid metabolism \|Lipid metabolism | 3.848122 | 0.014641 | 1.452923 |
| Sucrose | ABC transporters \|Galactose metabolism \|Carbohydrate digestion and absorption \|Taste transduction \|Starch and sucrose metabolism | Membrane transport \|Carbohydrate metabolism \|Digestive system \|Sensory system \|Carbohydrate metabolism | 1.025201 | 0.015234 | 1.980176 |
| Choline | Choline metabolism in cancer \|Glycerophospholipid metabolism ABC ransporters \|Cholinergicsynapse \|Glycine, serine and threonine metabolism \|Bile secretion | Cancers: Overview \|Lipid metabolism \|Membrane transport \|Nervous system \|Amino acid metabolism \|Digestive system | 2.255288 | 0.015803 | -0.31067 |
| L-Proline | Central carbon metabolism in cancer \|Protein digestion and absorption \|Biosynthesis of amino acids \|Aminoacyl-tRNA biosynthesis \|ABC transporters \|Arginine and proline metabolism \|Mineral absorption | Cancers: Overview \|Digestive system \|Global and overview maps \|Translation \|Membrane transport \|Amino acid metabolism \|Digestive system | 2.40119 | 0.016881 | -0.58186 |
| PC(14:1(9Z)/20:1(11Z)) | Choline metabolism in cancer \|Glycerophospholipid metabolism \|Linoleic acid metabolism \|Retrograde endocannabinoid signaling \|alpha-Linolenic acid metabolism \|Arachidonic acid metabolism | Cancers: Overview \|Lipid metabolism \|Lipid metabolism \|Nervous system\| Lipid metabolism\|Lipid metabolism | 6.063918 | 0.017205 | -2.11273 |
| PC(18:0/22:6(4Z,7Z,10Z,13Z,16Z,19Z)) | Choline metabolism in cancer \|Glycerophospholipid metabolism \|Linoleic acid metabolism \|Retrograde endocannabinoid signaling \|alpha-Linolenic acid metabolism \|Arachidonic acid metabolism | Cancers: Overview \|Lipid metabolism \|Lipid metabolism \|Nervous system\| Lipid metabolism \|Lipid metabolism | 5.079599 | 0.017607 | -2.51382 |
| PC(16:0/20:3(8Z,11Z,14Z)) | Choline metabolism in cancer \|Glycerophospholipid metabolism \|Linoleic acid metabolism \|Retrograde endocannabinoid signaling \|alpha-Linolenic acid metabolism \|Arachidonic acid metabolism | Cancers: Overview \|Lipid metabolism \|Lipid metabolism \|Nervous system\| Lipid metabolism \|Lipid metabolism | 4.052698 | 0.018615 | -1.59766 |
| SM(d18:0/24:0) | Sphingolipid signaling pathway \|Sphingolipid metabolism \|Necroptosis | Signal transduction \|Lipid metabolism \|Cell growth and death | 2.726028 | 0.019447 | 1.512818 |
| L-Methionine S-oxide | Cysteine and methionine metabolism | Amino acid metabolism | 1.428068 | 0.023656 | -0.47717 |
| Taurine | ABC transporters \|Primary bile acid biosynthesis \|Neuroactive ligand-receptor interaction \|Taurine and hypotaurine metabolism \|Sulfur metabolism | Membrane transport \|Lipid metabolism \|Signaling molecules and interaction \|Metabolism of other amino acids \|Energy metabolism | 1.081049 | 0.023764 | -0.45387 |
| PC(16:1(9Z)/18:1(9Z)) | Choline metabolism in cancer \|Glycerophospholipid metabolism \|Linoleic acid metabolism \|Retrograde endocannabinoid signaling \|alpha-Linolenic acid metabolism \|Arachidonic acid metabolism | Cancers: Overview \|Lipid metabolism \|Lipid metabolism \|Nervous system\| Lipid metabolism \|Lipid metabolism | 3.523202 | 0.024663 | -1.90109 |
| PC(18:1(11Z)/18:1(11Z)) | Choline metabolism in cancer \|Glycerophospholipid metabolism \|Linoleic acid metabolism \|Retrograde endocannabinoid signaling \|alpha-Linolenic acid metabolism \|Arachidonic acid metabolism | Cancers: Overview \|Lipid metabolism \|Lipid metabolism \|Nervous system\| Lipid metabolism \|Lipid metabolism | 4.725734 | 0.025554 | 0.592338 |
| PC(16:1(9Z)/18:1(11Z)) | Choline metabolism in cancer \|Glycerophospholipid metabolism \|Linoleic acid metabolism \|Retrograde endocannabinoid signaling \|alpha-Linolenic acid metabolism \|Arachidonic acid metabolism | Cancers: Overview \|Lipid metabolism \|Lipid metabolism \|Nervous system\| Lipid metabolism\|Lipid metabolism | 6.563836 | 0.030212 | -1.47107 |
| PE(22:2(13Z,16Z)/18:1(11Z)) | Glycerophospholipid metabolism \|Retrograde endocannabinoid signaling \|Glycosylphosphatidylinositol (GPI)-anchor biosynthesis \|Autophagy – other \|Autophagy – animal \|Kaposi sarcoma-associated herpesvirus infection | Lipid metabolism \|Nervous system \|Glycan biosynthesis and metabolism \|Transport and catabolism \|Transport and catabolism \|Infectious diseases: Viral | 1.093423 | 0.031975 | 0.68492 |
| PC(18:2(9Z,12Z)/18:1(11Z)) | Choline metabolism in cancer \|Glycerophospholipid metabolism \|Linoleic acid metabolism \|Retrograde endocannabinoid signaling \|alpha-Linolenic acid metabolism \|Arachidonic acid metabolism | Cancers: Overview \|Lipid metabolism \|Lipid metabolism \|Nervous system\| Lipid metabolism \|Lipid metabolism | 3.733152 | 0.035361 | -1.6511 |
| Hippuric acid | Phenylalanine metabolism | Amino acid metabolism | 1.172328 | 0.035591 | -1.02148 |
| LysoPC(20:3(5Z,8Z,11Z)) | Choline metabolism in cancer \|Glycerophospholipid metabolism | Cancers: Overview \|Lipid metabolism | 2.382791 | 0.043844 | -0.51417 |
| PC(20:0/14:0) | Choline metabolism in cancer \|Glycerophospholipid metabolism \|Linoleic acid metabolism \|Retrograde endocannabinoid signaling \|alpha-Linolenic acid metabolism \|Arachidonic acid metabolism | Cancers: Overview \|Lipid metabolism \|Lipid metabolism \|Nervous system\| Lipid metabolism \|Lipid metabolism | 3.251675 | 0.047915 | -1.3743 |
